# Rates and predictors of attrition among children on antiretroviral therapy in Ethiopia: A prospective cohort study

**DOI:** 10.1371/journal.pone.0189777

**Published:** 2018-02-06

**Authors:** Mulatu Biru, Inger Hallström, Pia Lundqvist, Degu Jerene

**Affiliations:** 1 Department of Health Sciences, Faculty of Medicine, Lund University, Sweden; 2 Management Sciences for Health, Addis Ababa, Ethiopia; National and Kapodistrian University of Athens, GREECE

## Abstract

**Introduction:**

Attrition from antiretroviral therapy (ART) programmes is a critical challenge among children receiving care in resource-limited settings. Our objective was to determine the rates and predictors of attrition among children on ART in Ethiopia.

**Methods:**

Between December 2014 and September 2016, we conducted a prospective cohort study in eight health facilities in Ethiopia. Eligibility criteria included age 3 months–14 years; being on ART for not more than a month. Outcome was attrition due to death and/or loss to follow-up. Predictor variables were child clinical and socio-demographic characteristics, and caregiver socio-demographic characteristics. We used Cox Regression analyses to examine the association between predictors and outcome.

**Results:**

Of 309 children, 304 were included, 52% were male. Their median age was 9 years (Inter-quartile range, IQR, 6–12). At ART initiation, their median CD4 was 362 cells/mm^3^ (IQR 231–499); and 74.3% had WHO stage 1 or 2 disease. During 287.7 person-years of observation (PYO), 24 attritions were recorded, yielding an attrition rate of 8.3 per 100 PYO (95% CI 5.4–12.1). Of these, six children were reported dead, leading to a mortality rate of 2.1 per 100 PYO (95% CI 0.8–4.3). Eighteen were lost to follow-up (LTFU) leading to LTFU rate of 6.26 per 100 PYO (95% CI: 3.83–9.70). The majority, 14 (58%) of attrition occurred during the first six months of treatment.

Age below three years [aHR] = 5.14 (95% CI: 2.07–12.96), rural residence (aHR = 3.97, 95% CI: 1.34–11.78) and baseline Hgb in g/dl < 10 g/dl [aHR] = 5.68 (95% CI: 2.03–6.23) predicted higher risk of attrition. Baseline Hgb < 10 g/dl (aHR = 16.63, 95% CI: 1.64–168.4) and WHO stage III or IV (aHR = 12.25, 95% CI: 1.26–119.05) predicted the death of the child. Higher attrition was documented among children of both biological parents alive and biologically related close family caregivers.

**Conclusion:**

Younger children, those from rural areas, and children with anaemia were at higher risk of attrition, especially during the early months of treatment, and therefore should be prioritized during treatment follow-up. Further studies should examine underlying reasons for higher attrition.

## Introduction

The wide-scale implementation of antiretroviral therapy (ART) averted the worst scenario of HIV transmission and related consequences among children living with HIV. Between 2000 and 2014, new HIV infections in children dropped by 58%, representing 220 000 fewer child infections per year, and a drop of 41% in sub-Saharan Africa [1]. Despite this impressive progress, half of the 1.8 million children living with HIV are still in need of ART [2].

In Ethiopia, the first adult HIV treatment guideline was published in 2003 but paediatric HIV care and treatment guideline was delayed until 2007 [3]. The ART coverage of 23.5% in children below 15 years of age is much lower than the 80% coverage reached in the adult population, indicating the unmet need of HIV treatment and care for children is very high in Ethiopia [4]. To address this huge gap, Ethiopia started a more ambitious accelerated action plan that to enrol 85% of children living with HIV for ART, retain 90% in ART and achieve viral suppression in 95% of children on ART [4]. This is aimed to improve paediatric HIV treatment, care and support [4, 5] in line with the goals of eliminating new paediatric infections by 2020 [2]. Moreover, the most recent national guidelines recommended the “test and treat all” strategy for all age groups [6] based on the new recommendations of the World Health Organization [7], further paving the way toward earlier initiation and thus prevention of death and further transmission of HIV.

Ensuring adequate retention in ART care is one of the critical steps needed to maximize the benefits of ART as a strategy to prevent death and disease transmission [8]. Studies characterizing rates and predictors of retention among paediatric patient groups will therefore serve as important inputs for countries to plan for appropriate interventions. However, a review of the current literature suggests that there are limited studies on this topic especially from resource-limited settings [9]. Moreover, most studies from these settings are retrospective in nature and there has been lack of standardization in the definitions of key outcome variables such as lost-to-follow-up (LTFU). In addition, outcomes of children reported to be LTFU were rarely reported and important potential predictors such as caregiver characteristics are often not included in the analysis.

A few studies from Ethiopia provided important insights about rates and predictors of attrition in children on ART [10–13]. However, none of these studies used a prospective study design. We previously reported our experience with non-adherence to ART during the early weeks of treatment [14]. In this paper, we describe results from longer-term follow-up of the same cohort of patients followed prospectively. The objective of this study was to determine rates and predictors of mortality and LTFU among children on ART in eight high-volume health facilities in the Addis Ababa and Oromia regions of Ethiopia.

## Methods

### Study design and setting

We conducted a prospective cohort study in eight health facilities in the Addis Ababa and Oromia regions of Ethiopia. The health facilities Addis Ababa were selected due to their high volume of children enrolled in ART and they serve patients coming with referral slips from all over the country. The selected health facilities in Oromia also serve a high load of adult and pediatric patients coming from urban and rural peripheries. Addis Ababa has a population of about 3.3 million and Oromia a total of about 29 million, accounting for 4.4% and 37% of Ethiopia’s population respectively in 2014 [15]. In Addis Ababa a total of 155 health facilities provide ART service, and 408 in Oromia [16].

### Participants and procedures

Children aged between 3 months and 14 years newly registered for ART between 20 December 2014 and 20 April 2015 in the participating health facilities were eligible for inclusion in the study. They were enrolled in the study within the first month of their ART treatment. Exclusion criteria included family caregivers of a child who has already been on ART for more than a month, a child with other severe illnesses or terminally ill and a child aged less than 3 months or over 14 years. OpenEpi statistical software was used for calculation of the sample size (http://www.openepi.com/Menu/OE_Menu.htm) applying the following assumptions: LTFU as the main outcome variable; inadequate family and heath care support as the exposure category (dichotomized as adequate or inadequate); per cent of exposed and unexposed groups with outcome as 30.6% and 15% respectively; Odds Ratio (OR) of 2.5; and power of 80% at 95% CI. The minimum sample size was expected to be 300 children including a 20% non-response rate.

According to the national guidelines, children returned for their first follow-up visit two weeks after ART initiation and every month thereafter for treatment refill and adherence assessment; and every three months for clinical examination and toxicity assessment [3]. During their visits to the ART clinic follow-up data were collected by an ART provider assisted by a data clerk who had received training in data collection and how to document data from the child’s medical records using a standardized and pre-tested data collection template. The first author (MB) provided follow-up training once or twice monthly and regular on-site support during data collection. The data collection template was developed in line with the national data capturing and documentation system.

We collected data on: socio-demographic and economic variables of family caregivers and children, clinical data of the child including baseline and progressive assessment of CD4 count, WHO clinical staging; child’s baseline treatment regimen and change of the treatment over the time, child’s baseline and after 12 months’ anthropometric measurements. The main outcome variable was attrition, defined as a combination of death and lost to follow-up as reported in patient records.

### Data analysis

We used the IBM Statistical Package for the Social Sciences (SPSS) version 22.0 for data entry and analysis (IBM Corporation, Armonk; NY, USA). Descriptive statistics present the child and caregivers characteristics in frequencies and proportion. Child’s Body mass index for age z-score (BAZ) was calculated using the AnthroPlus software available at www.who.int. BAZ with implausible values, as flagged by the macros, were excluded from the analysis. As the anthropometric z-scores calculation could be done for all ages and are able to measure underweight closely in all age groups, we included only BAZ in the analysis from the available anthropometric z-scores. Time duration was adjusted for the child’s heterogeneous enrolment in treatment. In our analysis, we used a comprehensive set of potential predictors including caregiver characteristics that were rarely used in earlier studies.

Baseline CD4 cell counts were collected and analysed based on the closest record to the date of treatment initiation during the period of two months before and one month after enrolment into ART initiation. Outcomes in the time period up to the end point of the 12-month follow-up were assessed by categorizing as under follow-up (retained in care), LTFU, dead (as recorded in treatment register) or transferred out (TO). Retention in care was defined as the proportion of children alive and still on ART 12 months after ART initiation and those TO, whereas attrition was defined as death and LTFU combined. Death was further ascertained through hospital admission report and caregivers’ report. LTFU was defined as a child whose treatment was interrupted for at least the first three months or more after the last visit date of the child.

We used WHO anaemia cut-off point to define anaemia based on the haemoglobin (Hgb) levels: children <5 years, 10–10.9 g/dl; 5–11 years, 11–11.4 g/dl and 12–14 years 11–11.9 g/dl [17]. Therefore, we took Hgb 10 g/dl to define anaemia in our dichotomized analysis as anaemia of <10 g/dl represents anaemia among all age groups of children.

We defined severe immunodeficiency based on the WHO classification of HIV-associated immunodeficiency in infants and children, which includes any child with the WHO stage IV or CD4% or CD4 count based on the child’s age, determined as CD4 < 25% or <1500 cells/mm^3^ for children aged ≤11 months; CD4 < 20% or <750 cells/mm^3^ in children aged 12–35 months; CD4 < 15% or <350 cells/mm^3^ in children aged 36–59 months; and CD4 < 15% or <200 cells/mm^3^ in children aged ≥5 years [18].

Attrition, LTFU and death during the follow-up were estimated using Kaplan–Meier methods. A cox proportional hazards model was used to assess independent predicting factors of an outcome. Multivariable model was used to adjust for age, sex, residence (rural, urban) CD4 cell count, WHO stage and Hgb g/dl. Variables with p < 0.25 in the univariate analyses were included in the multivariate analyses. Body mass index for age (BAZ) and severe immunodeficiency were adjusted to control the possibility of confounding in predicting death. Statistical significance was described as adjusted hazard ratio (HR) with 95% CI. Statistical significance was set at p < 0.05.

## Ethical considerations

The study was approved by the Swedish Regional Ethics Board (Ref. no. 2013/85), Addis Ababa University College of Health Science Institutional Review Board (Protocol no. 045/13/sph) and the National Research Ethical Review Committee (Ref. no. 3.10/084/2015) in Ethiopia. The international principles of research ethics outlined in the Declaration of Helsinki were strictly followed [19]. Informed consent was obtained from all caregivers for their children to be included in the study. Oral explanation about the purpose, possible benefits and disadvantages of the study and the right to withdraw at any time was provided to the participants. In addition, the ART clinic focal person also signed as an impartial witness. Confidentiality was assured. None of the authors were involved in the care of the children.

## Results

### Baseline results

**Fig 1:**
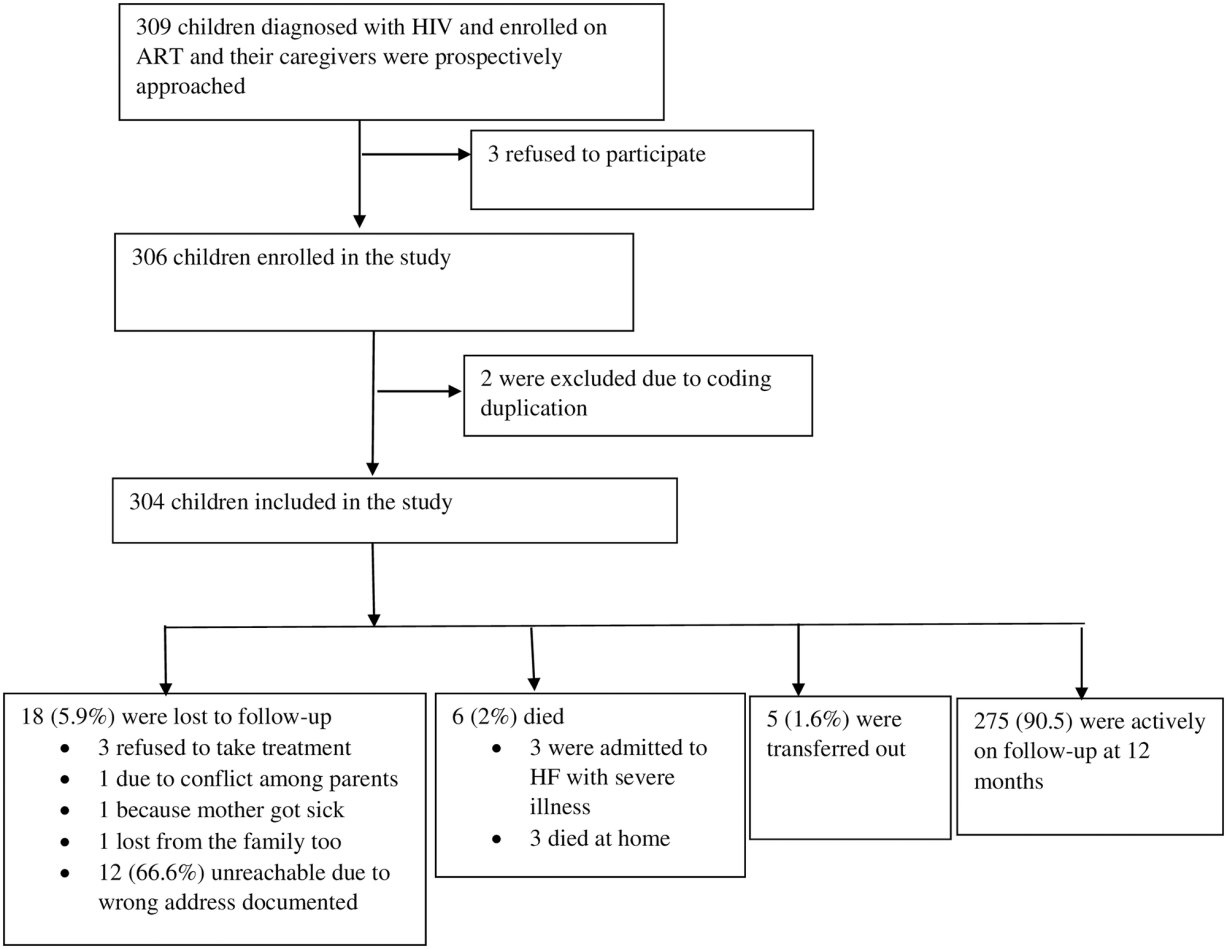
Flow chart showing the cohort profile.

Caregivers of 309 potentially eligible children were approached; three caregivers refused to allow their children to participate in the study, making the response rate 99%. Two children were found to have the same unique code and were excluded from the follow-up pool, resulting in 304 children on follow-up. Fig 1 shows the cohort profile.

The distribution of girls and boys was 48.4% and 51.6%, with the median age of 9 years for both and interquartile range, IQR, 6–13 and 5–12 respectively. At ART initiation, the median CD4 count for girls and boys was 365 cells/mm^3^ (IQR 244–504) and 358 cells/mm^3^ (IQR 229–499) and median BMI for age z-score were –0.86 (IQR –1.8–0.005) and –0.75 (IQR –1.4–0.1) respectively. At ART initiation, 226 (74.3%) had WHO stage 1 or 2 disease, of whom the majority 119 (52%) were boys; and 11% were started on a protease inhibitor-based regimen, of whom 53% were girls (Table 1). At baseline, 212 (70%) of children were started on the first-line regimen of AZT-3TC-NVP/EFV; 31 (10%) received D4T-3TC-NVP/EFV; 23 (7.6%) received ABC-3TC-NVP/EFV/KAL; and 38 (12.5%) received triple fixed dose combination (FDC) TDF-3TC-NVP/EFV. Table 1 describes baseline characteristics of the study participants.

**Table 1:** Baseline characteristics of study participants, Ethiopia.

Characteristics	Number	%
Sex		
Female	147	48.4
Male	157	51.6
Age median (IQR) years	9 (6–12)	
0–3	35	11.5
4–8	71	23.4
> 9	198	65.1
WHO stage at enrolment		
Stage I or II	226	74.3
Stage III or IV	78	25.7
Baseline ARV		
Other	272	89.5
PI-based*	32	10.5
Base line CD4 count cells/mm^3^ [median (IQR)]	362 (231–499)	
Female	365 (IQR 244–504)	
Male	358 (IQR 229–499)	
BMI for age z-score [median (IQR)]	–0.8 (–1.6–0.04)	
Female	–0.86 (IQR –1.8–0.005	
Male	–0.75 (IQR –1.4–0.1)	
Caregiver’s sex		
Female	249	82
Male	55	18
Caregiver’s age, years		
Median (IQR)	36 (30–41)	
18–24	18	6
25–34	125	41
35–44	109	36
> 45	52	17
Marital status of caregiver		
Married	160	53
Widowed/divorced	98	32
Single	46	15
Educational status of caregiver		
Illiterate	51	17
Literate (read & write)	31	10
Grades 1–8 completed	108	36
High School completed	84	28
College/University level	30	10
Caregivers Residence		
Rural	25	8
Urban	279	92
Caregiver-child relationship		
Biological parents	223	73
Non-biological caregivers	81	27

*PI = protease inhibitor (Lopinavir + ritonavir) or Kaletra based

### Follow-up results

The cohort contributed 287.7 person-years of observation (PYO) with the median (IQR) follow-up being 0.99 (IQR 0.87–1.08) years. At the end of follow-up, 90.5% of the children were under active follow-up while 24 attritions were recorded yielding an attrition rate of 8.3 per 100 PYO (95% CI 5.4–12.1) and most of the attrition 14 (58%) occurred during the first six months of treatment. Eighteen (75%) of the attrition was due to LTFU while death accounted for the remaining 6 (25%). The LTFU rate was thus 9.12 per 100-PYO. Upon further tracing, three of those LTFU were due to refusal to take the treatment; three were due to conflict between parents; caring mother was sick for one child and one child was lost from the family as well. The remaining 66.6% (12/18) were unreachable due to wrong telephone and residence address documented in child’s record.

The overall mortality rate was 2.1 per 100 PYO (95% CI 0.8–4.3) and most of the deaths (five out of six) occurred during the first eight months of follow-up. Possible underlying causes of death were ascertained for three out of six deaths; two died after hospital admission due to opportunistic infection and one child died due to severe anaemia. The cause of death for the remaining three was not recorded as these deaths occurred at home.

At twelve months of follow-up, the majority 208 (68.4%) of the children were still on the first-line regimen of AZT-3TC-NVP/EFV/KAL, 48 (16%) received ABC-3TC-NV/EFV/KAL and 48 (16%) received TDF-3TC-NVP/EFV.

### Predictors of attrition

**Fig 2:**
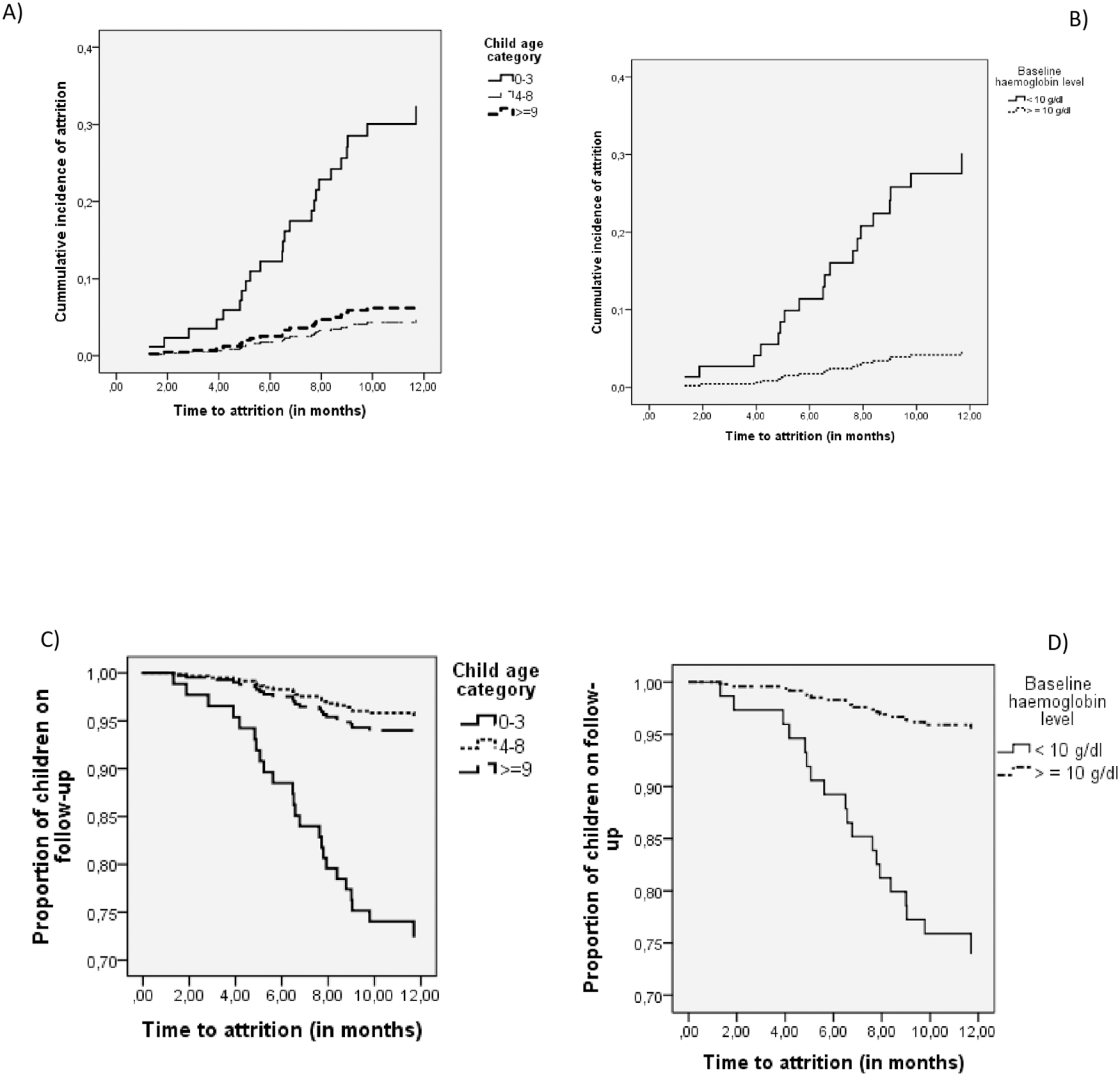
Cumulative incidence and survival curve of recorded attrition. A) Cumulative incidence by child age group, B) Cumulative incidence by child baseline Hgb, C) Survival curve by child age group and D) Survival curve by child baseline Hgb.

In the univariate analyses, factors significantly associated with child’s attrition were child age below three years, caregivers of the children coming from rural areas and baseline Hgb < 10 g/dl. Fig 2 shows the Kaplan-Meier survival and cumulative incidence curves.

In multivariate analysis, child age below three years (aHR = 5.3, 95% CI: 1.62–17.24), caregivers of the children coming from rural areas (aHR = 3.97, 95% CI: 1.34–11.78) and baseline Hgb < 10 g/dl (aHR = 3.3, 95% CI: 1.03–10.8) were found to be significantly associated with child’s attrition as shown in Table 2. Moreover, coming from rural areas (aHR = 3.57, 95% CI: 1.10–11.64) and age below three years (aHR = 3.76, 95% CI: 1.16–12.27) were found to predict the LTFU of the child (Table 3).

**Table 2:** Hazard ratios (HR) of attrition according to Cox regression analyses, Ethiopia, 2017.

Hazard ratios (HR) of Attrition						
Variable	Participants	Incident Attrition	Person-Year	Attrition rate per 100 PYO	cHR (95%CI)	aHR (95%CI)
Hgb ^b^						
<10 g/dl	41	10	35.5	28.57 (13.68, 52.55)	6.59 (2.67, 16.24)*	3.3 (1.0, 10.8)*
> = 10 g/dl	216	9	207.75	4.33 (1.975, 8.214)	Ref	
Severe Immunodeficiency ^a^						
Yes	74	6	68.77	8.82 (3.222, 19.21)	0.41 (0.13, 1.35)	0.62 (0.23. 1.66)
No	230	18	218.92	8.25 (4.89, 13.05)	Ref	Ref
Child Age (years)						
<3	35	9	29	3.10 (1.51, 5.69)	4.84 (2.04, 11.51)*	5.3 (1.62, 17.24)*
3–8 yrs	71	3	67.69	4.47 (1.14, 12.19)	0.69 (0.19, 2.45)	0.56 (0.15, 2.05)
> = 9	198	12	191	6.28 (3.40, 10.68)	Ref	Ref
Caregivers’ Residence						
Rural	25	5	22.22	22.52 (7.25, 52.51)	3.12 (1.16, 8.35)*	3.97 (1.34, 11.78)*
Urban	279	19	265.5	7.15 (4.307, 11.18)	Ref	Ref
Baseline WHO stage						
I And II	226	17	214.5	7.93 (4.614, 12.69)	0.82(0.34, 1.99)	
III and IV	78	7	72.78	9.62 (3.854, 19.82)	Ref	
Baseline child BMI-for age						
z-score < -2	42	6	38.35	15.65 (5.71, 34.05)	2.47 (0.96, 6.38)	
z-score > 2	253	15	241.9	6.20 (3.46, 10.23)	Ref	
Child Sex						
Female	147	12	141.1	8.51 (4.393, 14.87)	1.02 (0.46, 2.28)	
Male	157	12	146.3	8.23 (4.242, 14.36)	Ref	
Biological Parents’ vital status						
Both parents alive	169	17	158.5	2.43 (1.41, 3.88)	1.96 (0.82, 4.74)	1.04 (0.34, 3.17)
One or both died	135	7	129.2	1.23 (1.04, 1.43)	Ref	Ref
Caregiver-child relation						
Biological family	239	21	225.04	9.3 (5.77, 14.26)	1.94 (0.58, 6.5)	1.07 (0.25, 4.49)
Others	65	3	62.65	4.79 (0.96, 13.99)	Ref	Ref

cHR = crude hazard ratio; aHR = adjusted hazard ratio;

^a^ CD4 counts were available for 291 (96%) children at enrolment;

^b^Baseline Hgb available for 257 (85%) children at enrolment.

* P value < 0.05

**Table 3:** Hazard ratios (HR) of LTFU according to Cox regression analyses, Ethiopia, 2017.

Hazard ratios (HR) of LTF						
Variable	No. Participants	Incident LTFU	Person-Year	LTFU rate per 100 PYO	cHR (95%CI)	aHR (95%CI)
Sex						
Female	147	7	141.1	4.96 (1.98, 10.22)	0.65 (0.25, 1.68)	
Male	157	11	146.3	7.52 (3.74, 13.45)	Ref	
Caregivers Residence						
Rural	25	4	22.22	18 (4.84, 46.09)	3.38 (1.11, 10.27)*	3.57(1.10, 11.64)*
Urban	279	14	265.47	5.27 (2.88, 8.84)	Ref	Ref
Age (in years)						
0–3	35	7	29	2.41 (1.05, 4.77)	5.61 (2.03, 15.51)*	3.76 (1.16, 12.27)*
4–8	71	3	67.36	4.45 (1.13, 12.12)	1.03 (0.28.3.92)	0.87 (0.22, 3.37)
> 9	198	8	190.99	4.19 (1.94, 7.95)	Ref	Ref
Baseline child BMI for age						
z-score < –2	42	3	38.35	7.82 (1.57, 22.86)	1.43 (0.41, 5.03)	1.37 (0.35, 5.29)
z-score > –2–2	253	13	241.93	5.37 (2.85, 9.18)	Ref	Ref
Baseline WHO stage						
stage I & II	226	15	214.6	6.99 (3.91, 11.53)	1.70 (0.49, 5.87)	1.36 (0.36, 5.06)
stage III & IV	78	3	72.77	4.12 (0.82, 12.04)	Ref	Ref
Biological Parents vital status						
Both parents alive	169	13	158.5	1.06 (0.91, 1.24)	2.09 (0.75, 5.89)	1.28 (0.37, 4.38)
One or both died	135	5	129.2	1.05 (0.87, 1.23)	Ref	Ref
Caregiver-Child relation						
Biological family	239	16	225	7.11 (4.06, 11.55)	2.21 (0.51, 9.59)	1.20 (0.22, 6.52
Others	65	2	62.65	3.19 (0.35, 11.53)	Ref	Ref

* P value < 0.05

On the other hand, baseline Hgb < 10 g/dl (aHR = 16.63, 95% CI: 1.64–168.4 and WHO stage III & IV (aHR = 12.25, 95% CI: 1.26–119) were found to predict the death of the child (Table 4). Higher rates of attrition documented among children of both biological parents alive and close biological relation of the caregiver (mother, father, brother or sister) compared to their counterparts.

**Table 4:** Hazard ratios (HR) of Death according to Cox regression analyses, Ethiopia, 2017.

Hazard ratios (HR) of Death						
Variable	No. Participants	Incident Death	Person-Year	Death rate per 100 PYO	cHR (95%CI)	aHR (95%CI)
Child age (in years)						
0–3	35	2	29	6.89 (1.15, 22.80)	3.29 (0.60, 18.01)	2.08 (0.29, 15.01)
4–8	71	0	67.4		0.000	0
> 9	198	4	191	2.09 (0.66, 5.05)	Ref	Ref
^a^Severe immunodeficiency						
Yes	74	2	68.44	2.92 (0.32, 10.55)	1.64 (0.30, 8.97)	0.92(0.15, 5.75)
No	230	4	218.9	1.83 (0.49, 4.67)	Ref	Ref
Baseline WHO stage						
stage I & II	226	2	214.58	0.93 (0.10, 3.36)	Ref	Ref
stage III & IV	78	4	72.78	5.50 (1.47, 14.07)	5.89 (1.08, 32.17)	12.25 (1.26, 119.05)*
^b^Baseline Hgb in g/dl						
< 10 g/dl	41	4	35.35	113.2 (30.45, 289.7)	2.42 (2.70, 216.7)	16.63 (1.64, 168.4)*
> = 10 g/dl	216	1	208.75	4.79 (0.06, 26.65)	Ref	

cHR = crude hazard ratio; aHR = adjusted Hazard ratio;

^a^CD4 counts were available for 291 (96%) children at enrolment;

^b^Baseline Hgb available for 257 (85%) children at enrolment.

* P value < 0.05

## Discussion

In this study, we found that during the first year of children’s enrolment in ART, the cumulative incidence of attrition was 8.3%, with LTFU accounting for three-fourths of the attrition and death contributing the remaining one-fourth. Most of the attrition occurred during the early months of follow-up. Younger children, those with baseline anaemia and age below three years were at significantly higher risk of attrition.

Our findings confirm the importance of the child’s age [9, 10, 13] and presence of baseline anaemia [9, 12] as reliable predictors of attrition from HIV care in a setting with limited resources. Furthermore, children enrolled in HIV care and treatment in our study facilities and coming from rural areas due to their caregivers’ residence were found to have greater risk of attrition than those from urban settings, which we identified as an additional caregiver-associated predictor of attrition. Other caregiver-related factors with higher rates of attrition include the existence of both biological parents and close biological relation of the caregiver with the child, which we report for the first time from Ethiopia and perhaps as one of a few studies globally. Our findings suggest the need to prioritize and provide tailored clinical care, counselling and support to children with the identified risk factors. In addition, caregiver characteristics should be taken into consideration while planning for more comprehensive care for children on ART.

In our study, the cumulative incidence of death is similar to that in previous studies in Ethiopia, but LTFU is lower than a recent report where the cumulative incidence of attrition 12 months after ART initiation was 15.5% [10]. Likewise, a comparison study of children enrolled in care in a public hospital and health centres in Ethiopia reported 34% LTFU and 2% death, adding up to an attrition of 36%. In previous studies, the attrition rate was found to be even higher among children enrolled in pre-ART care. The five-year pre-ART follow-up study among children in India has reported attrition rate of 16% and a 17.9% LTFU was reported after one year in Ethiopia [10, 20, 21]. The possible reason for the lower attrition rate in our study could be partly be the prospective follow-up and regular reminders to the caregivers through ART providers and data clerks. The prospective follow-up in our study has also most likely avoided an overestimation of LTFU, by reducing misclassification of unknown or undocumented transfers out and deaths as LTFU, which is an inherent limitation of retrospective studies [10]. In addition, most of the study facilities have institutionalized a new strategy of peer support groups, aimed to promote treatment adherence and child’s retention in care and treatment. Furthermore, greater emphasis has been placed on paediatric antiretroviral treatment and care regardless of CD4 count or clinical stage, in line with the 2013 WHO consolidated guideline [22, 23].

Regardless of lower attrition rate compared to previous studies, our study indicates that a considerable number of children were still LTFU from care. Among those lost to follow-up after enrolment in ART, 78% and 50% were LTFU before six and four months, respectively. This is evidence along with previous studies that synergetic effort is required particularly in resource-limited settings (RLS) including: creating a conducive environment at health facilities for children including shortening the waiting time, issuing an electronic reminder to enhance child’s medication adherence, creating a joint network between health facility adherence support unit and community health workers to perform active and early tracing of missed appointments. Having such a coordinated action in place would greatly enhance retention on treatment and overall favourable treatment outcome for all children in HIV care, [14], while there is limited evidence of a single effective strategy to promote retention in RLS [10]. In this study attrition rate tended to be greater among children age 3 years and younger, which confirms results from other large-scale studies carried out among children in sub-Saharan Africa, India and Ethiopia [10, 20, 24].

Children with lower Hgb levels (< 10 g/dl) had greater risk of attrition, mainly due to higher risk of death. ARV treatment initiation, particularly with Zidovudine (AZT), could worsen existing anaemia in children, predisposing further to opportunistic infections and eventual death. Children with baseline haemoglobin levels below 10 g/dl should be given special attention and close follow-up while initiating ART [3].

Although we had quite few participants in the study, children coming from rural areas have greater risk of attrition, which could be because they have a longer distance to a treatment facility, lack of unaffordable transportation, and lack of caregiver to accompany them to the clinic visit, indicating the need to ensure both accessibility and provision of quality ART care service for children at their nearby clinics. Children with advanced clinical stage upon initiation of ART were at the highest risk of death, highlighting the need for early treatment initiation irrespective of CD4 count [9, 12]. Surprisingly, higher rates of attrition which mainly derived from LTFU were found among children of both biological parents alive and biologically related caregivers. The possible reason for this could be stigma and discrimination against people with HIV in the studied areas, which is also in line with the evidence we reported in our earlier study [14]

Lack of baseline viral loads as a predictor variable was the main limitation of this study. However, the use of prospective follow-up design along the course of treatment is believed to have increased the completeness of recording and minimized misclassification of outcomes, which is the main strength of this study. Other strengths in this study include the high response rate of participants in the follow-up, the attempts we made to trace all of the LTFU children to determine the final outcome status, and availability of information about the places and causes of death.

### Conclusions

Attrition during the first year after ART initiation was dependent on several factors including child and caregiver individual and socio-demographic characteristics. Younger children, those from rural areas and those with anaemia were at significantly higher risk of attrition. In addition, the advanced stage of disease at baseline and baseline Hgb were found to predict the death of the child. Furthermore, higher rates of attrition were documented among children of both biological parents alive and biologically related close family caregivers.

Our findings highlight the need to give more attention to families of children with the identified risk factors as part of the care and treatment package for children on ART. In addition, there is a need to pay greater attention to children at greater risk of death due to having lower blood haemoglobin and having developed advanced disease at treatment initiation. As the ART scale-up become universal, there is great need for focused interventions to ensure retention and minimize attrition caused by death or LTFU. While the overall attrition rate is low compared with earlier reports, further studies are needed to better understand the underlying reasons for the attrition. Longer-term follow-up of the cohort will give further insights into how the attrition rate will change over the years.
